# Early Identification of Central Retinal Artery Occlusion Using Point-of-care Ultrasound

**DOI:** 10.5811/cpcem.2018.11.39406

**Published:** 2019-01-04

**Authors:** Ben Stoner-Duncan, Stephen C. Morris

**Affiliations:** University of Washington School of Medicine, Department of Emergency Medicine, Seattle, Washington

## Abstract

A 69-year-old woman with a history of untreated hypertension presented with acute-onset monocular vision loss. Initial workup was delayed due to lack of immediate specialty consultation and dilated funduscopic exam. Point-of-care ultrasound in the emergency department identified a small hyperechoic structure within the distal area of the central retinal artery; in conjunction with specialty ophthalmologic evaluation in a tertiary care center, the diagnosis of central retinal artery occlusion was made. The patient was admitted to the neurology service for stroke risk stratification and was discharged in stable condition with re-initiation of her antihypertensive medication regimen.

## INTRODUCTION

Painless vision loss represents a small proportion of emergency department (ED) visits but can portend not only an acute threat to vision, but also significant systemic pathology. Thromboembolic disease related to cardiovascular or stroke risk factors,^1^ rheumatologic disease such as giant cell arteritis, or pathology localized to the eye can all present in a similar fashion.^2^ For many causes of acute painless vision loss, dilated fundoscopic exam is considered the gold standard for diagnosis; however, this can be a challenging exam in some patients, especially in resource-limited settings without expert consultation available. It has previously been shown that point-of-care ultrasound (POCUS) can be used to reliably evaluate arterial and venous flow in cases of retinal vessel compromise using color Doppler imaging,^3^ and to directly visualize retinal artery thrombus.^4^ Here we present a case of central retinal artery occlusion (CRAO) identified by POCUS, with evidence of edematous optic nerve, a previously undescribed ocular ultrasound finding in the evaluation of CRAO.

## CASE REPORT

A 69-year-old woman presented to the ED with painless vision loss in her left eye. She stated that over the course of minutes, she completely lost vision in that eye, with onset approximately six hours prior to evaluation. Initially, there was concern for posterior circulation arterial stroke, as the patient had elected to stop taking her antihypertensive medications one year prior. A computed tomography (CT) of the head showed no evidence of intracranial hemorrhage, and a magnetic resonance imaging (MRI) evaluation of the brain showed no evidence of acute stroke. The patient’s fundoscopic exam was limited by constricted pupils, and she was transferred to a tertiary care academic medical center for ophthalmologic evaluation.

On arrival to the tertiary care center, the ED team performed a POCUS, linear probe, 12 MegaHertz (MHz) of the patient’s eye to evaluate for retinal detachment. No evidence of retinal detachment, vitreous detachment, or massive vitreous hemorrhage was found. However, the study demonstrated a widened and irregular optic nerve sheath, which measured over the normal limit of five millimeters (mm).^5^ Additionally, an area of hyperechoic signal was noted in the distal aspect of the optic nerve, raising concern for embolic event (Image). Additional radiologist review of the patient’s MRI showed no evidence of embolism in that area. Ophthalmology was consulted and performed a dilated fundoscopic exam, with direct visualization of a pale, occlusive object within the central retinal artery.

The patient was admitted to the neurology service for monitoring of permissive hypertension initially, and then resumption of an antihypertensive medication regimen. The timing of symptom onset was a contraindication for thrombolytic treatment. Rapid stroke-risk stratification demonstrated no echocardiographic evidence of cardiac source of her embolus, no right-to-left cardiac shunt, and no significant carotid stenosis on CT angiography. She was re-initiated on her outpatient antihypertensive medications, and on two-month follow-up she had no significant return of vision in her left eye and had no evidence of further embolic events.

## DISCUSSION

POCUS is of increasing utility to the emergency physician. The optic globe, as a superficial fluid-filled object, lends itself to reliable ultrasound imaging with potential diagnostic utility in a number of acute ophthalmologic emergencies.^6^ Here we present an instance of CRAO identified rapidly on POCUS. Multiple prior studies have reported on ultrasonographic findings related to CRAO. A prior study of 29 patients diagnosed with CRAO, but without evidence of retinal plaque on fundoscopy, revealed that 31% showed retrobulbar hyperechoic foci on orbital color Doppler imaging.^3^

In another study, 59% of patients with CRAO were found to have an “intra-arterial spot sign,” which was associated with lack of response to thrombolytic therapy. This lack of response is thought to be primarily due to calcific components of likely carotid thromboembolic plaque, which is not amenable to thrombolysis.^7^ Elucidating cardiovascular risk factors associated with retinal artery occlusions, 79.8% of patients with CRAO had hypertension, as did our patient. Although dilated funduscopic exam reveals pale macula and a “cherry red spot” in the vast majority of cases, only 11% of CRAO thrombi were directly visualized fundoscopically.^1^ Given the possibility of diagnostic uncertainty and delay in specialty consultation, this indicates that POCUS may offer a more rapid diagnosis of this important entity and could also aid in diagnostic reliability.

The finding of edematous optic nerve may provide another reproducible finding, though this has not been well described in the literature. A measurement pattern has been established to have a sensitivity of 88% and specificity of 93% for optic nerve sheath edema due to intracranial pressure of greater than 20 centimeters water based on an optic nerve sheath diameter of greater than 5mm.^5^ In our reported case, it is biologically plausible that this finding was related to time after occlusion, and may be able to provide some evidence of the timing of occlusion onset in cases with an unclear “last known normal.” This measurement, combined with previously described Doppler studies may also offer increased sensitivity for rapid POCUS diagnosis. More rapid diagnosis in cases of CRAO allows for possible interventions such as ocular massage, thrombolysis, surgical intervention to lower intra-ocular pressure and earlier initiation of stroke workup in order to reduce patient risk. Future studies should focus on reliability of optic sheath measurement in reaction to CRAO, and occurrence of ultrasound-evident occlusive emboli in these cases.

**CPC-EM Capsule**

What do we already know about this clinical entity?

*These findings have been well documented in other imaging sources, particularly magnetic resonance imaging. Use of ultrasound has been reported and quantified but is not widely disseminated in the literature*.

What makes this presentation of disease reportable?

*The image gives a guide to practicing physicians as to appearance of the edematous optic nerve*.

What is the major learning point?

*Incorporating ultrasound in the evaluation and management of patients presenting with vision loss could potentially speed the diagnosis of central retinal artery occlusion*.

How might this improve emergency medicine practice?

*Emergency physicians reading this article would be able to incorporate ultrasound in evaluation and management of patients presenting with vision loss, potentially speeding the diagnosis of central retinal artery occlusion*.

## CONCLUSION

Central retinal artery occlusion is a rare presentation to the ED; however, it represents significant pathology, with concerns for long-term disability (vision loss) and possible life-threatening associated conditions (stroke.) Use of point-of-care ultrasound represents an opportunity to make a rapid and accurate diagnosis of CRAO.

## Figures and Tables

**Image:**
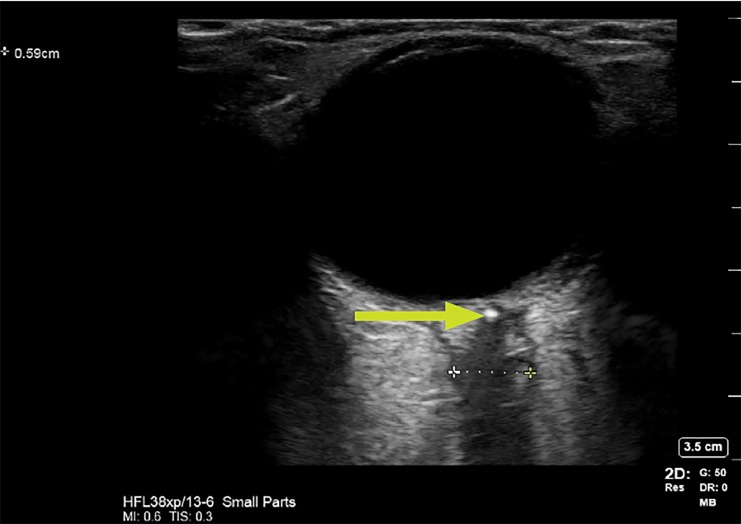
Point-of-care ultrasound of left eye with linear probe small-parts setting, demonstrating likely clot (yellow arrow) and edematous optic nerve (measured at 3.5cm).
